# Randomized controlled trial of vitamin d supplementation on toll-like receptor-2 (tlr-2) and toll-like receptor-4 (tlr-4) in tuberculosis spondylitis patients

**DOI:** 10.1186/s13018-023-04445-6

**Published:** 2023-12-21

**Authors:** Jainal Arifin, Muhammad Nasrum Massi, Karya Triko Biakto, Agussalim Bukhari, Zairin Noor, Muhammad Phetrus Johan

**Affiliations:** 1 Orthopaedic and Traumatology Department, Faculty of Medicine, Hasanuddin University, Makassar, Indonesia; 2 Clinical Microbiology Department, Faculty of Medicine, Hasanuddin University, Makassar, Indonesia; 3 Clinical Nutrition Department, Faculty of Medicine, Hasanuddin University, Makassar, Indonesia; 4 Orthopaedic and Traumatology Department, Faculty of Medicine, Lambung Mangkurat University, Banjarmasin, Indonesia

**Keywords:** Vitamin D, TLR-2, TLR-4, Tuberculosis spondylitis

## Abstract

**Background:**

Tuberculosis spondylitis accounts for approximately 50% of all cases of skeletal tuberculosis. Vitamin D plays a role in the immune system. Vitamin D helps in the activation of TLR-2 and TLR-4, which play a role in the process of tuberculosis infection. The objective of this study was to investigate the effect of oral supplementation with vitamin D on TLR-2 and TLR-4 levels in tuberculosis spondylitis patients.

**Methods:**

The true Experiment Design Pretest–Posttest with Control Group (Pretest–Posttest with Control Group) was used for this research. TLR-2 and TLR-4 were measured by ELISA. Repeated ANOVA, ANOVA tests, and Kolmogorov–Smirnov normality tests on the SPSS program were used to statistically analyze the results.

**Result:**

In the dose groups of 10,000 IU and 5000 IU, significant increases in the levels of vitamin D, TLR-2, and TLR-4 were observed at weeks 4 and 8 (*p *< 0.05). In the control group, there was no significant increase.

**Conclusions:**

Vitamin D supplements can significantly increase TLR-2 and TLR-4 levels. Supplementation with vitamin D 10,000 IU/day for 8 weeks can increase vitamin D levels > 50 ng/dl to optimally act as an immunomodulator.

## Introduction

Tuberculosis (TB) spondylitis is a spinal infectious disease caused by the bacterium *Mycobacterium tuberculosis*, an enzyme-producing bacterium that is highly aerobic, and acid-resistant not contain proteolytics [1]. TB spondylitis represents approximately 50% of all cases of skeletal tuberculosis. People with disorders of the immune system due to chemotherapy in cancer patients, elderly individuals, diabetes mellitus, alcoholism, malnutrition, drug abuse, and patients with HIV are observed [1, 2]. Vitamin D deficiency has always been associated with an increased risk of infection with *Mycobacterium tuberculosis* [3]. In the study, Tang et al. [4] found that vitamin D deficiencies correlated with spondylitis tuberculosis type necrosis.

Vitamin D is a prohormone that plays an important role in the absorption of calcium in the intestines [5, 6]. Vitamin D, in its active form 1,25-hydroxyvitamin D, has a complex activity in the immune system by inducing and inhibiting a process to destroy MTB [7]. Vitamin D itself enters the immune system against MTB in general through several mechanisms, among other things: fagolisosome, as revealed by Chocano-Bedoya P. The important role of vitamin D in immune response includes producing LL-37, which will induce fagolisosomes against MtB [8]. Both autophagi and vitamin D-mediated innate immunity were revealed by Yuk et al., [9] where 1,25-dihydroxyvitamin D induces the occurrence of autophagy in monocytes. Autophagy and vitamin D-mediated innate immunity can provide protection against *Mycobacterium tuberculosis* infection and prevent bacteria from causing further damage. Increased nitrogen oxide response (NO) and reactive oxygen species (ROS), as revealed by Yang et al. [10] Vitamin D can enhance the function of nitrogen oxide (NO) and reactive oxygen species (ROS) in eliminating Mycobacterium tuberculosa. As revealed by Wahyunitisari et al. [11], Vitamin D regulates caspase-mediated apoptosis. This is programmed cell death without causing an inflammatory reaction. Vitamin D is useful in the treatment of tuberculosis.

Toll-like receptors (TLRs) is a family of proteins that are expressed either on the surface of extracellular cells, in the cytosol, or on the endosome membranes of macrophages and dendritic cells. TLRs plays a key role in the innate immune response to infectious agents [12]. Among the many TLRs that play a role in the body as a congenital immune system against MTB bacteria, TLR 2 and 4 are the most studied and most closely associated with *Mycobacterium tuberculosis* [12].

Several mycobacterial antigens such as LpqH, lipoarabinomannan (LAM), lipomannan (LM), 38-kDa antigen, LprG, LprA, PhoS1, trehalose dimycolate (TDM), and phosphatidylinositol mannoside (PIM) will activate TLR2 [13]. The distribution of TLR2 on various types of cells, e.g., immune, endothelial, and epithelial cells, is observed. The activation of TLR2 signaling benefits the host defense against invading pathogens [14]. The TLR2 agonist LpqH from *Mycobacterium tuberculosis* activates apoptosis and autophagy in macrophages, which are pathways that inhibit intracellular *Mycobacterium tuberculosis* growth [15]. Activation of TLRs by mycobacterial antigens leads to intracellular signaling pathways that culminate in the production of pro-inflammatory factors in macrophages and dendritic cells [13]. Meanwhile, TLR4 can recognize pathogen-associated molecular patterns (PAMPs) [5] and induce the release of inflammatory cytokines, such as TNF-*α*, IL-6, IL-1*β*, and IFN-*γ*. *Mycobacterium tuberculosis* presents type of PAMP, liposaccharides (LPS) [16].

## Methods

**Fig. 1:**
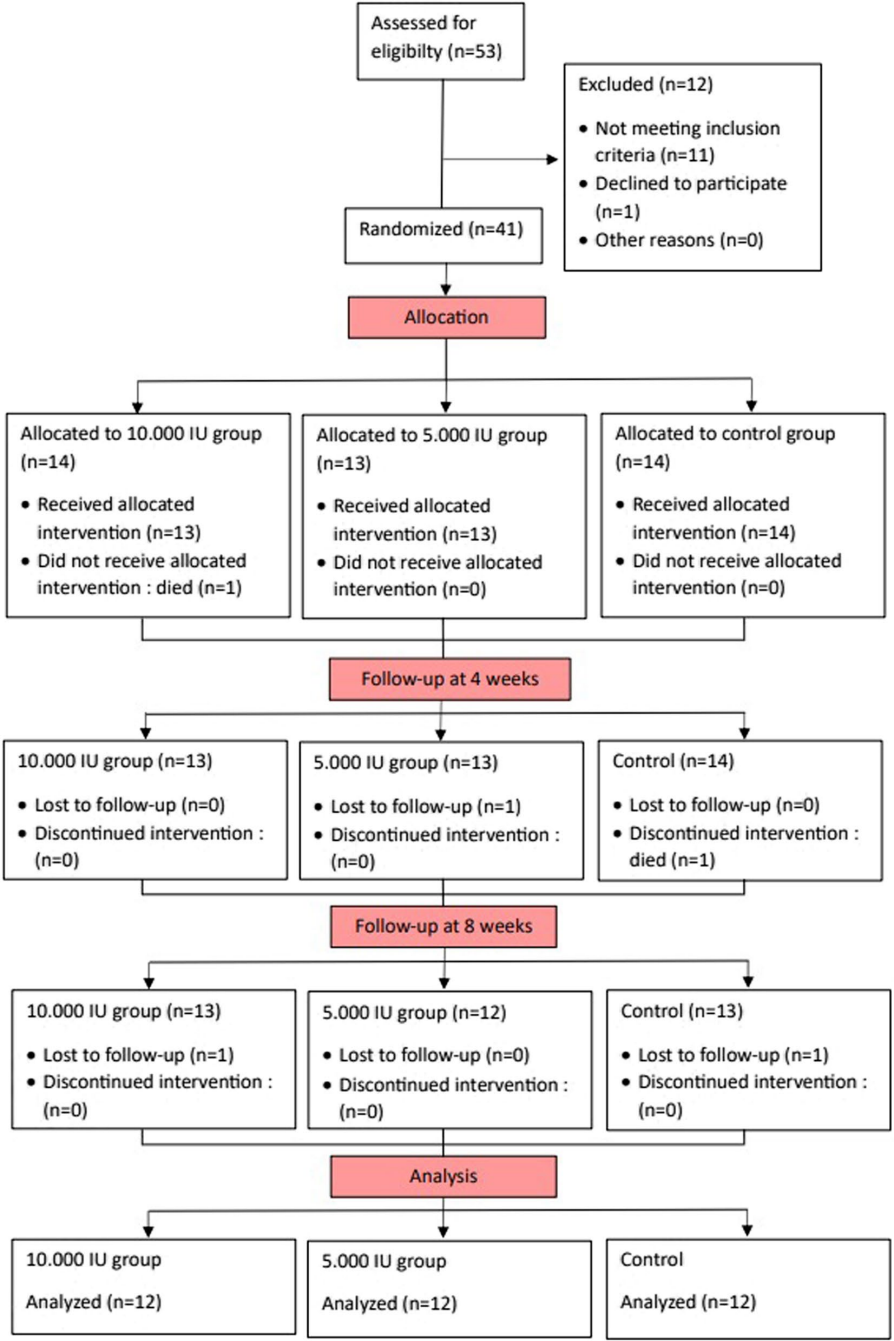
Flowchart

The research design was the true Experiment Desain Pretest–Posttest dengan Grup Kontrol (Pretest–Posttest with Control Group) which examined the effect of vitamin D supplementation on TLR2 and TLR4 as well as clinical outcomes measured before and after administration of vitamin D supplementation. This research was conducted from July 2022 to December 2022. Subjects were selected by a convenience random sampling method. The total number of subjects found was 53 participants who were assessed for eligibility in this study. However, 11 participants did not meet the inclusion criteria, and one participant declined to participate in this study because of refusal to have blood samples collected. The inclusion criteria were TB patients aged 19–50 years, who were diagnosed with hematology, MRI, and GenExpert. Subjects had to sign a consent form. The exclusion criteria were pulmonary tuberculosis, other extrapulmonary tuberculosis, and any chronic disease (renal failure, diabetes, hypertension, autoimmune liver, cancer, alcoholism malabsorption syndrome). (Fig. 1).

Before supplementation, 41 subjects met inclusion criteria and agreed to involve in this study. Three participants were excluded because they died in the middle of the study and two participants were excluded due to loss to follow-up. All the subjects were divided into three groups. The study group received vitamin D supplementation at 10,000 IU/day and 5,000 IU/day, and the control group received 400 IU/day. Oral vitamin D supplementation was started concurrently with anti-TB drugs. A total of 36 participants were treated and completed with supplementation and blood sampling; 12 participants were placed in the vitamin D dose group of 10,000; 12 participants were included in the vitamin D dose group of 5000 as a study group; and 12 participants were placed in the 400-dose vitamin D group as a control group. (Fig. 1).

Blood samples were collected after 4 and 8 weeks of supplementation. Three milliliter of venous blood samples were collected from the subjects. The sample was immediately centrifuged for 10–15 min to separate the blood serum. The blood serum was put into Eppendorf tubes and stored in a freezer at –20◦C and taken to the laboratory. The samples were processed for ELISA analysis. TLR2 and vitamin D concentrations were recorded in ng/ml, and TLR4 was recorded in pg/ml.

All reagents were incubated at room temperature (18–25 °C) before use. After that, 30 mL of concentrated wash buffer was diluted with 720 mL of deionized or distilled water to prepare 750 mL of wash buffer. The standard will be centrifuged at 10,000 × g for 1 min. The sample diluent and 1.0 mL of reference standard were allowed to stand for 10 min and inverted gently several times. Each well was filled with 100 μL standard or sample and incubated for 90 min at 37 °C. The liquid was removed, and 100 μL of biotinylated detection was added. The sample was incubated for 1 h at 37 °C and washed three times. HRP Conjugate 100 μL was added. The samples will be incubated for 30 min at 37 °C. Then, the sample was washed five times. Substrate reagent (90 μL) was added and incubated for 15 min at 37 °C. The stop solution (50 μL) was added. Then, all the samples will be read at 450 nm in an ELISA microplate reader.

Statistical analysis was performed with SPSS v.23 using a descriptive data normalization test and bivariate analysis of numerical data. Kolmogorov–Smirnov ANOVA test, repeated ANOVA test, and Pearson test. The statistical test is significant if the *p* value < 0.05.

## Results

The mean age of the participants was 36.0 ± 13.2, with total participants having a percentage of males (52.8%) and females (47.2%), a body mass index less than 18, and a body weight of 48.7 ± 8.6. Neurological examination assessments in participants found normal motor (50%) and normal sensory functions (47.2%). The data were then tested using the Kolmogorov–Smirnov method to determine the distribution of data normality. The test results showed normally distributed data (*p *> 0.05).

**Fig. 2:**
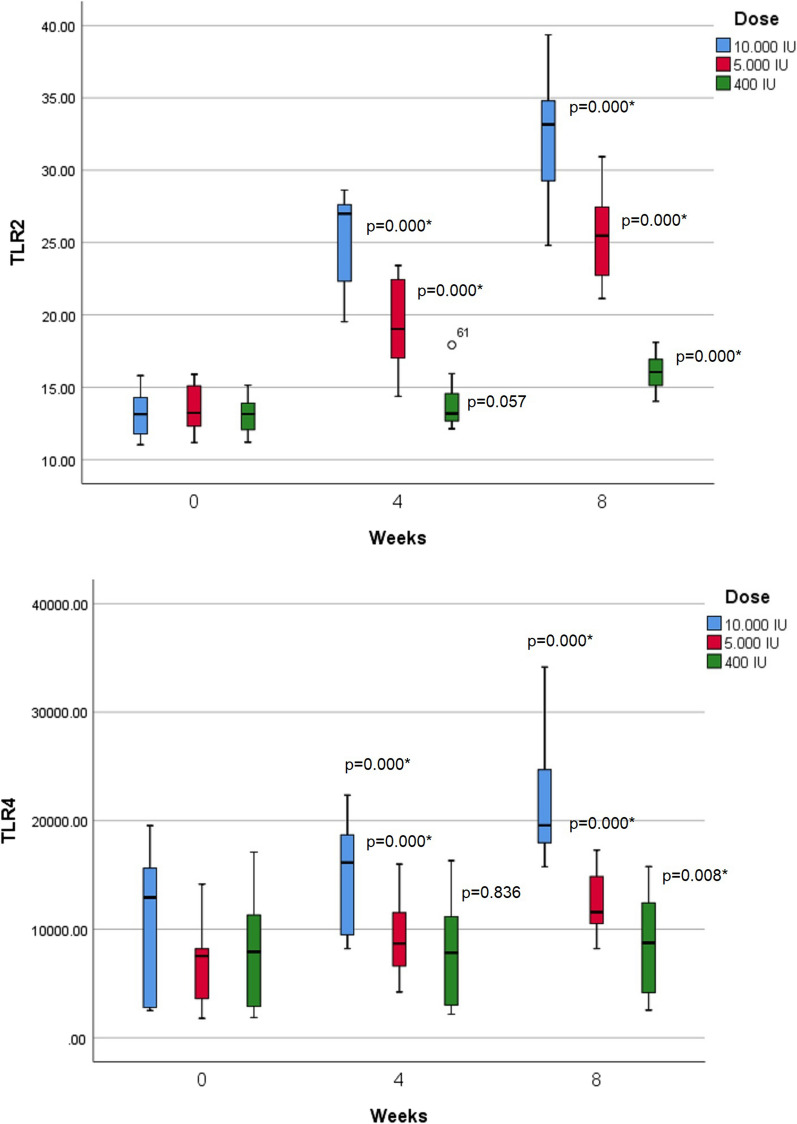
The concentration of TLR-2 and TLR-4

In the comparison of each group before and after supplementation, obtained at week 4, the difference in TLR-2 levels at doses of 400 IU and 5,000 IU was not significant. At week 8, there were significant differences in TLR-2 levels between the groups (Fig. 2). In trials using repeated ANOVA, the three dosing groups of 10,000 IU and 5000 IU experienced significant increases from week 0 to weeks 4 and 8 (*p *< 0.05). In the 400 IU dose group, there was no significant increase from week 0 to week 4 (*p *= 0.057).

The difference in TLR-4 levels at doses of 400 IU and 5000 IU was not significant. At week 4, there was a significant difference in TLR-4 levels in the 10,000 IU dose group versus the other groups. (Fig. 2). In trials using repeated ANOVA, the three dosing groups of 10,000 IU and 5000 IU experienced significant increases from week 0 to weeks 4 and 8. In the 400 IU dose group, there was no significant increase from week 0 to week 4 (*p *= 0.836).

After 4 and 8 weeks of intervention, there were significant differences in vitamin D levels in the three groups. In the 400 IU dose group, the vitamin D levels did not increase significantly through repeated ANOVA from week 0 to week 4 (from 28.38 ± 6.51 ng/ml to 27.62 ± 6,54 ng/mL, = 0.76, *p *= 1,000), and from week 4 to week 8 there was no significant increase. Vitamin D levels in the 400 IU group from week 0 to week 8 did not increase significantly (from 28.38 ± 6.51 ng/ml to 30.04 ± 4.15 ng/mL, = 1.65, *p *= 0.786). In the 400 IU dose group, vitamin D levels were decreased at week 4. In the 5,000 IU and 10,000 IU dose groups, there was a significant increase from week 0 to weeks 4 and 8 (Fig. 2).

## Discussions

All groups experienced increased vitamin D levels at both week 4 and week 8. The 10,000 IU dose group saw the highest increase in vitamin D during 8 weeks of supplementation, from 27.91 ± 8.68 ng/ml to 66.40 ± 4.89 ng/mL. Supplementation of vitamin D 10,000 IU/day for 8 weeks can increase vitamin D levels > 50 ng/dl so that it functions optimally as an immunomodulator. Meanwhile, at other doses it did not reach > 50 ng/ml.

A study conducted by Nurpudji et al. [17] showed an increase in vitamin D levels after the administration of a dose of 10,000 IU for 2 weeks. In the study carried out by Salma et al. [18], increased vitamin D levels were found after vitamin D supplementation. This study showed significant increases in vitamin D levels at weeks 4 and 8 in the group given vitamin D supplementation compared to the placebo group. Vitamin D can decrease due to several factors, such as dark skin color, the use of sunscreen, and UV exposure. In addition, decreased production of vitamin D can also be caused by geographical factors such as: season, latitude, air pollution, fog/clouds, and humidity. Absorption disorders can also affect the decrease in vitamin D such as celiac disease, pancreatic disorders, and obstruction of the biliary tract [6, 19].

Vitamin D deficiency has always been associated with an increased risk of infection with *Mycobacterium tuberculosis* [20] In a study, Tang et al. [4] found that vitamin D deficiencies correlated with spondylitis tuberculosis type necrosis.

A study by Gibney et al. [21] showed lower levels of vitamin D in patients with latent TB infection than in healthy people. Increased vitamin D can affect the low probability of latent tuberculosis infection. In addition to vitamin D, another study conducted by Orafli et al. [22] found that vitamin A deficiency also plays a role in tuberculosis infection in the Moroccan population. Previous research also found no effect of high doses of vitamin A on the outcome of children with tuberculosis infection. Vitamin A has also been shown not to affect sputum conversion in patients with pulmonary tuberculosis infection. Patients with pulmonary tuberculosis infection who were given OAT treatment found that their vitamin A levels would return to normal even though they were not given supplementation [23]. However, in a study conducted by Visser et al. [24], it was found that supplementation with vitamin A combined with zinc for 8 weeks was considered to have no effect on the outcome of patients with pulmonary tuberculosis infection.

**Fig. 3:**
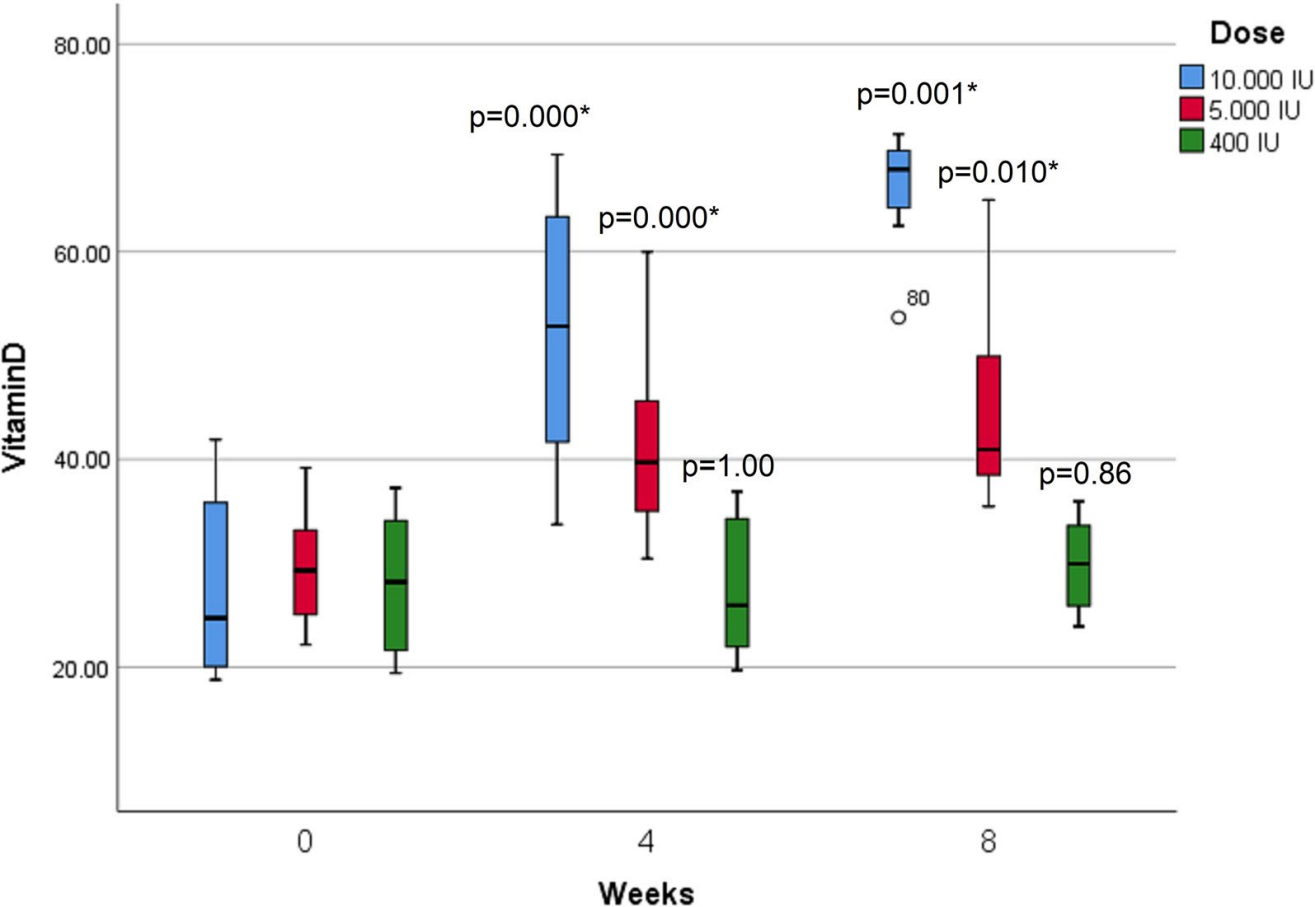
The concentration of vitamin D

Vitamin D supplementation at doses of 400, 5000, and 10,000 mg/dl for 8 weeks may also affect TLR 2 and TLR 4 levels. There was a difference between TLR 2 and TLR-4 levels of 7 mg/dl to 15 mg/dL at week 8 compared to week 0. The study also found a significant correlation between vitamin D levels for TLR-2 and TLR-4. After supplementation, TLR-2 and TLR-4 levels increased. This increase in TLR indicates an increased immune response that plays a role in the process of fighting *M. pneumoniae* and tuberculosis. TLR activation by the Mycobacterium antigen will give the cell a response to produce more pro-inflammatory cytokines in macrophages and dendritic cells [13] (Fig. 3, Table 1).

**Table 1:** Characteristics of the subjects

Characteristic	Total (%)	10,000 IU N (%)	5,000 IU N (%)	400 IU N (%)	*p* value
Number of participants	56	12	12	12	
*Gender*					
Male	19 (52.8)	7 (58.3)	6 (50.0)	6 (50.0)	
Female	16 (47.2)	5 (41.7)	6 (50.0)	6 (50.0)	.895
Age	36.0 ± 13.2	34.6 ± 14.5	42.0 ± 12.1	30.9 ± 8.7	.491
*Motoric*					
Normal	18 (50.0)	6 (50.0)	8 (66.7)	4 (33.3)	
Parese	9 (25.0)	2 (16.7)	3 (25.0)	4 (33.3)	
Plegia	9 (25.0)	4 (33.3)	1 (8.3)	4 (33.3)	
*Sensoric*					
Normal	17 (47.2)	4 (33.3)	8 (66.7)	5 (41.7)	
Hypoesthesia	14 (38.9)	8 (66.7)	3 (25.0)	3 (25.0)	
Anesthesia	5 (13.9)	0 (0.0)	1 (8.3)	4 (33.3)	

Simultaneous activation of the TLR2 and TLR4 signaling pathways induces macrophage apoptosis and induction of apoptosis and necrosis in response to *Mycobacterium tuberculosis* [25]. Mycobacterial components are recognized by TLR-2 and TLR-4. Several mycobacterial antigens including lipoarabinomannan (LAM), 38-kDa antigen, LpqH, lipomannan (LM), LprG, PhoS1, LprA, phosphatidylinositol mannoside (PIM), and trehalose dimycolate (TDM) activate TLR2, TLR1, or TLR6, whereas TLR4 recognizes 38-kDa and 60/65 heat shock protein (HSP) antigens. TLR 2 and TLR-4 receptors are overexpressed during infection [13, 26]. Activation of TLRs by antigens belonging to mycobacteria leads to intracellular signaling pathways that culminate in pro-inflammatory production in dendritic cells and macrophages through the MAPK and NF-κB pathways [10]. In a study by Wani et.al, TLR-4 (Thr/Ile) and TLR-2 (Del/Del) acted as significant risk factors for extrapulmonary tuberculosis in the ethnic Kashmiri population [27] Salih et al. [28] revealed that the upregulation of TLR- 2 and TLR-4 due to single nucleotide polymorphisms (SNPs) may be involved in the tuberculosis infection process in Sudanese individuals.

In a study conducted by Ali et al. on TLR-2 levels in cases of tuberculosis infection, significant differences in serum TLR2 levels were found in each variation of TB infection cases. In cases of recurrence of tuberculosis infection, TLR-2 serum levels were found to decrease significantly compared to new cases of TB infection [29]. In another study conducted by Hendri et al., it was found that vitamin D levels correlated positively with TLR2 serum expression in systematic lupus erythematosus patients. The higher the levels of vitamin D, the higher the TLR-2 expression in the saliva sample examined [30]. In a study conducted by Eve et al. [20], vitamin D supplementation was found to increase TLR2 levels in pregnant women. Hendri et al. [31] found that vitamin D levels correlated with serum TLR-4 expression in systematic lupus erythematosus patients.

Other studies showed similar results, as Rahmini Shabariah et al. showed a significant increase in serum levels of TLR-2 and TLR-4 in patients with extrapulmonary TB who had post-vaccination scars of *Mycobacterium bovis* Bacillus Calmette–Guerin (BCG) compared to patients without BCG scars. The formation of scars after BCG vaccination affects the formation of immunity against M infection. TLR-2 and TLR-4 serum scores were higher in patients with extrapulmonary tuberculosis, in addition to serum TLR-2, TLR-4 was higher in those with BCG scars than in those without BCG, and statistical analysis results showed a significant difference. Additionally, evidence suggests that toll-like receptor 2,4 (TLR-2 and TLR-4) is associated with susceptibility to TB by interacting with toll-like interactive protein (TOLLIP) to activate macrophages [32].

Vitamin D has functions in the immune system, cell proliferation, and differentiation. Supplementation of vitamin D to > 100 nmol/L shows an increase in TLR-2 expression and a decrease in the TLR2-profile of cytokine stimulation for TNF, IL-6, and IFN, and this condition will be reversed when vitamin D doses are reduced to < 100 nmol/L (*p *= 0.002). Optimal vitamin D supplementation can improve TLR-2 expression and increase the body’s ability to fight infections [27].

The side effect of supplementation with vitamin D is intoxication. It is characterized by markedly elevated vitamin D concentrations (usually > 150 ng/ml), hypercalcemia, hypercalciuria and PTH suppression. Clinical manifestations are associated with hypercalcemia and include: weakness, fatigue, confusion, drowsiness, apathy, vomiting, constipation, polyuria, polydipsia, and abnormalities on the electrocardiogram (reduced Q-T interval) [33]. However, in this study, no participant had an intoxication of vitamin D. TLR-4 has other important roles that can be activated by mycobacterial lipopolysaccharides and lipotechoid acids, one of the two immune pathways that the inhibitor can activate through TLR-4. TLR-2 and TLR4 are risk factors for extrapulmonary TB [27].

## Conclusion

Increased TLR-2 and TLR-4 levels indicate an increased immune response that plays a role in the fight against *M. tuberculosis* infection. Vitamin D supplementation at dosage of 10,000 IU/day increased TLR expression. Vitamin D supplementation can improve TLR expression and increase the body’s ability to fight infections. Supplementation with vitamin D 10,000 IU/day for 8 weeks can increase vitamin D levels > 50 ng/dl to optimally act as an immunomodulator. From this study, it can be concluded that there is a significant influence of vitamin D on levels of TLR-2 and TLR-4. Patients with tuberculosis spondylitis infection are advised to receive vitamin D supplementation at a dose of 10,000 IU as an additional therapy with OAT to eliminate mycobacterium tuberculosis. The authors recommend further research on the effect of vitamin D on macrophages with microbiological examination and examination of other biomarkers. The study should also distinguish between primary and latent infection.

## Data Availability

Not applicable.

## Abbreviations

TB: Tuberculosis
TLR: Toll-like receptor
ODI: Oswestry disability index
VAS: Visual analog scale
